# Cross-cultural adaptation and validation of the reintegration to normal living index into IGBO language among individuals with mobility disability

**DOI:** 10.1186/s41687-019-0139-9

**Published:** 2019-07-12

**Authors:** Emmanuel Chiebuka Okoye, Stella Onyinye Oyedum, Christopher Olusanjo Akosile, Ifeoma Uchenna Onwuakagba, Peter Olanrewaju Ibikunle, Uchenna Prosper Okonkwo, Ifeoma Adaigwe Okeke

**Affiliations:** 1 Department of Medical Rehabilitation, College of Health Sciences, Nnamdi Azikiwe University, Nnewi Campus, Anambra State, 435101 Nigeria; 2 Department of Physiotherapy, Nnamdi Azikiwe University Teaching Hospital, Nnewi, Anambra State, Nigeria

**Keywords:** Disability, Reintegration to Normal living index, Igbo culture and environment, Cross-cultural adaptation, Validation

## Abstract

**Background:**

Community reintegration is one of the most important elements of disability rehabilitation globally. Hence, there is need for availability of psychometrically-sound and culturally-specific instruments for its measurement. Most of the available community reintegration measures were developed and validated in developed countries and might therefore not be suitable for use in developing countries. This study was aimed at cross-culturally adapting and validating the original English visual analogue scale version of the Reintegration to Normal Living Index (RNLI) into Igbo Language and culture among people with mobility disability in Igbo land, Southeast Nigeria. The English version of the RNLI was cross-culturally adapted to Igbo following the American Association of Orthopaedic Surgeons’ guideline. The RNLI was translated into Igbo Language, synthesized, back translated, and subsequently subjected to expert panel review, pretesting and cognitive debriefing interview. The final Igbo version of the RNLI was tested for internal consistency and construct validity in a sample of 102 consenting participants (61.8% males; 46.92 ± 20.91 years) recruited from conveniently sampled clinics and rehabilitation centres in Anambra and Enugu States of South-Eastern Nigeria. The construct (concurrent) validity was evaluated using Spearman rank correlation, scatter plot and Mann-Whitney U test while the internal consistency was evaluated using Cronbach’s alpha at alpha level of 0.05.

**Results:**

The RNLI was successfully cross-culturally adapted to Igbo with all the 11 items still retained. The mean total score of the participants on the RNLI was 58.62 ± 21.25. The internal consistency coefficient (α = 0.84) of Igbo version of the RNLI was excellent. The Spearman correlation coefficients between the participants’ total, subscale and domain scores on the Igbo and the English versions of the RNLI (r = 0.81–0.95) were excellent. There was no significant difference between corresponding scores in the English and Igbo versions of the RNLI.

**Conclusion:**

The Igbo version of the RNLI is a valid and reliable outcome measure among Igbo people living with mobility disabilities in Southeast Nigeria. It is therefore recommended for use among this group.

## Background

Disability, an umbrella term for impairments, activity limitations and participation restrictions, is an issue of global concern [1, 2]. More than one billion people in the world (15% of world population) live with some form of disability, of which nearly 200 million experience considerable difficulties in functioning [2]. The prevalence of disability is very high in developing countries with about 40% of African population consisting of people with disability [2]. In Nigeria, about 14 million out of a total population of over 140 million were with disability [3]. The prevalence of disability has been projected to increase in the years ahead due to increase in aging population and chronic health conditions such as diabetes, cardiovascular disease, cancer and mental health disorders [2]. People with disabilities (especially in less advantaged communities) have poorer health outcomes, lower educational achievements, less economic participation, higher rates of poverty, higher medical expenses, less community participations and integration than people without disabilities [2; 4]. Mobility disability is the most frequently reported disability type [5] with nearly 40% of adults over age 45 having difficulty with physical movement [6].

Mobility disability results in varying degrees of limitation in aspects of a person’s physical functioning, and can be caused by respiratory, orthopaedic or neuromuscular disorders such as cerebral palsy, stroke, muscular dystrophy, spinal cord injuries/paralysis, back conditions, arthritis, severe cases of repetitive strain injury (RSI), fibromyalgia, myalgic encephalomyelitis/chronic fatigue syndrome, multiple sclerosis, and cystic fibrosis [7]. Mobility disability may result in the sufferer being restricted in: the use of one or more extremities; the ability or stamina to stand or walk independently over short distances; the ability to be adequately physically active; and the strength or dexterity to grasp or lift objects [7, 8]. People with mobility disabilities might use mobility aids to get around, such as a wheelchair, scooter, rollator/walker, or crutches, or they might require personal care assistance for essential aspects of daily living such as feeding, dressing, washing, and toileting [7]. All these limitations can restrict the sufferers’ participation and integration/reintegration into his/her community [2, 4].

Community reintegration is commonly defined as the opportunity an individual has to live in the community with the already present condition/ill-health/disability and be valued for his/her uniqueness and abilities, like everyone else [9]. It has attracted considerable attention in rehabilitation of sufferers of chronic conditions [10]. Community reintegration is the most important and ultimate goal of any rehabilitation programme but ironically the most underestimated area [11]. The goal of rehabilitation has shifted from mere survival and improvement in physical, psychological and social health to how well a sufferer of a chronic debilitating condition is reintegrated/integrated into their community [10, 12–14]. Community integration and community reintegration are synonymous and are usually used interchangeably in literature [9, 15, 16]. It is a multidimensional construct which definition, perception and components vary and differ across authors, settings, target populations, groups, age groups, cultures, environments, races, et cetera [12, 17, 18]. The benefits of community integration are reportedly numerous and include physical, social, psychological, health, and quality of life related outcomes [19].

The concept of community reintegration is reportedly culture- and environment-specific as its meanings and interpretations can vary with races, groups, disabilities, age groups, social roles and cultures [15, 17]. From empirical observations, these variations in the components and interpretations of community reintegration are more obvious when comparing developed and developing countries as their cultures and environments vary markedly. These variations can be seen in: building and living arrangements; popular means of transportation; recreational activities; social activities; work life; family arrangement and roles; et cetera. Consequently, there are needs for availability of culture- and environment-specific tools for assessment of community integration during rehabilitation [13, 14].

Many tools for assessing community integration abound in literature [10, 20–25]. They include but are not restricted to: the Craig Handicap Assessment and Reporting Technique; the Reintegration to Normal Living Index (RNLI); the Community Integration Questionnaire; the Subjective Index of Physical and Social Outcome; the Community Integration Measure; and the Maleka Stroke Community Reintegration Measure. However, all these tools (except the Maleka Stroke Community Reintegration Measure that was developed in South-Africa) were initially developed and validated for assessing community integration in high income countries of the world. Consequently, these tools may not be entirely suitable for use in low/middle income countries thus making proper assessment of community integration in these countries difficult [26, 27]. Even though the Maleka Stroke Community Reintegration Measure was developed and validated for use in low/middle income countries (including Nigeria) [28, 29], its use is restricted to only stroke survivors.

When the problem of unavailability of culture-specific outcome measures is encountered, stakeholders in health are usually faced with two options: development of new tools for each culture and environment or cross-cultural adaption of the existing tools to suit the culture and environment of interest. However, it is always advisable to cross-culturally adapt an existing scale in a different culture and environment as against developing a new one; as the former is more economical than the latter, and can facilitate comparisons among populations [26, 30]. For quality control during the process of cross-cultural adaptation, standardized protocols for translation and cross-cultural adaptation of questionnaires were developed. The American Association of Orthopaedic Surgeons’ guideline developed by Beaton et al. [26] is a widely accepted protocol that involves two forward translations, synthesis, two backward translations, expert panel review, pretesting, cognitive debriefing interview and validation. The different stages of this protocol were geared towards reducing bias as much as possible during the process.

Apart from the stroke-specific Maleka Stroke Community Reintegration Measure, no other tool for assessment of community integration has been cross-culturally adapted and validated for use in a Nigerian environment. For better cost-effectiveness and enhanced future utility, cross-culturally adapting a generic as against a disease-specific scale may be a better bet. Among the aforementioned community integration tools, only the Craig Handicap Assessment and Reporting Technique and the RNLI are generic [28]. However, when compared with the Craig Handicap Assessment and Reporting Technique, the RNLI is easier to administer, has been more widely used in literature, and has been translated into more languages [14, 20, 31–34]. The present study was therefore designed to translate, cross-culturally adapt and validate the RNLI among Igbos with mobility disabilities. Igbo Language is one of the three major native languages in Nigeria (the most populous black nation) and a minor language in Equatorial Guinea, with about 24 million speakers [35].

## Methods

This is a validation study that employed the American Association of Orthopaedic Surgeons’ guidelines for cross-cultural adaptation developed by Beaton et al. [26]. An approval to carry out the research was obtained from the Ethics Committee of Nnamdi Azikiwe University Teaching Hospital, Nnewi before the commencement of the study. Permission to translate the original English version of the RNLI (E-RNLI) was obtained from the developers [20]. Three tertiary hospitals and two rehabilitation centres in Anambra and Enugu States of the South-Eastern (Igbo land) Nigeria were conveniently selected for this study. The centres were Nnamdi Azikiwe University Teaching Hospital, Nnewi, Anambra State; Chukwuemeka Odumegwu Ojukwu University Teaching Hospital, Awka, Anambra State; University of Nigeria Teaching Hospital, Ituku-Ozalla, Enugu State; Salvation Army Rehabilitation Centre Oji River, Enugu State; and Leprosy Colony Oji River, Enugu State. The participants were adults (18 years and above) who verbally acknowledged significant restriction in their mobility. The participants’ mobility restrictions were caused by variety of ailments including amputation, stroke, spinal cord injury, severe osteoarthritis, rheumatoid arthritis, post-polio syndrome, osteogenesis imperfecta, leprosy, neuropathy, fracture or foot drop for a period of at least three months. The participants could understand both Igbo and English Languages. Individuals who were in-patients, not well-oriented in time, place and person, or those who could not respond to the questionnaires were excluded from the study. The eligibility criteria were applicable for participants in both the adaptation (phase-2) and validation (phase-3) phase of the study. Each participant signed or thumb-printed the consent form after the nature and objectives of the study had been duly explained to them. The socio-demographic variables of age, gender, level of formal education, marital status, employment status and period of living in the community of the participants were recorded.

### Instruments for data collection

#### The RNLI

This is a generic tool developed in Canada for assessing perceived reintegration to normal living (community reintegration) after an incapacitating illness [20]. It is a descriptive and evaluative tool that can be administered as an interview or can be filled in by the clients on their own or can even be administered by proxy. It can equally be administered on telephone. It is an 11-item tool with the following domains: indoor mobility, community mobility, distance mobility, self-care, daily activities (work and school), recreational activities, social activities, family roles, personal relationships, presentation of self to others, and general coping skills [20, 36]. The first eight items constitute the “daily functioning” subscale while the last three items make up the “perception of self” subscale [37]. The RNLI has many scoring systems (including 4-point ordinal scale, 3-point ordinal scale, 10 cm visual analogue scale, 10-point Likert scale and a dichotomous response scale (yes/no)) [14] but the 10 cm visual analogue scale is the most commonly used. Generally, visual analogue scale responses has been previously reported to display better validity, reliability and responsiveness coefficients when compared with multi-response options [38]. Consequently, the visual analogue scale form of the RNLI was cross-culturally adapted and validated in the present study. In the visual analogue scale format of the RNLI, each item is accompanied by a 10 cm visual analogue scale with 0 signifying no integration (does not describe my situation) and 10 signifying full integration (fully describe my situation) [39]. A total score is obtained by summing up the individual item scores. The total score is then normalized to 100 such that the minimum and maximum possible scores are 0 and 100, indicating no and full integration respectively. The questionnaire can take about 10 min to administer and about 5 min to score. It has been translated into other languages, and has been reported to have excellent validity and reliability scores and high utility [14, 20, 31, 32].

### Cross-cultural adaptation and validation of the RNLI

The procedure employed in this study followed the American Association of Orthopaedic Surgeons’ guidelines for cross-cultural adaptation developed by Beaton et al. [26] as recommended by the original developers of the RNLI [14]. The procedure for the study was in three phases: Phase 1-translation phase, Phase 2-adaptation phase and Phase 3-validation phase.

#### Phase 1 -- translation phase

This involves forward (initial) translation, synthesis of the translation and back translation. The forward translation involved the translation of the E-RNLI into Igbo language. Two bilingual Translators whose mother tongue was Igbo language produced two different Igbo translations (T1 and T2). The two translators had different backgrounds. Translator 1, a physiotherapist experienced in translating questionnaires, was aware of the concepts of the questionnaire being translated (community reintegration). The primary aim of this translation was to provide equivalence from a more clinical perspective and also to produce a translation providing a more reliable equivalence from a measurement perspective. Translator 2, a linguist without medical or clinical background, was neither aware nor informed about the concepts being quantified. Translator 2 was meant to offer a language translation as used by the population, often highlighting ambiguous meanings in the original questionnaire. The two translations (T1 and T2) were then synthesized by the two translators and a recording observer. Working from the original questionnaire as well as the two translated versions (T1 and T2), a synthesis of these translations was then conducted (producing one common translation T-12), with a documented report carefully describing the process of synthesis. The synthesized T-12 version of the RNLI was totally translated back to the English language by two independent bilingual translators (producing two English versions BT1 and BT2) who were unaware and informed of the concepts being explored. Back translators were an experienced physiotherapist translator and a physiotherapy undergraduate who were very fluent in both Igbo and English Languages. Back translation was for checking the validity of the questionnaire so as to ensure that the translated version was reflecting the same item content as the original version. Two back-translated English versions (B1 and B2) were thus produced.

#### Phase 2 -- adaptation phase

The E-RNLI and the forward (T1, T2, T12) and the back translations (BT1 and BT2) were reviewed by a panel of experts so as to produce a version expertly adapted to Igbo culture and environment. The expert committee comprised the four translators (forward and backward translators), three physiotherapy researchers (excluding one of the forward translators), an outcome methodologist, and a lay person (an executive officer in a University). Members of the expert panel were very familiar with the Igbo culture and environment. Discrepancies in the translations were resolved by consensus in order to achieve semantic equivalence, idiomatic equivalence, experiential equivalence and conceptual equivalence of the pre-final Igbo version of the questionnaire. The pre-final Igbo version of the questionnaire was subjected to field testing by administering it to 30 participants [26] with mobility disabilities (who met the inclusion criteria) after informed consent had been sought and obtained from the participants. One of the authors also took the 30 participants through cognitive debriefing interview which involved determination of what each participant thought was meant by each questionnaire item and the responses. The lead author queried the participants on the following: ease of understanding each item; if there is ambiguity in each item; if the response items for each item are difficult to understand; if the activity depicted in each item was being practiced in Igbo culture; and if the participants thought that all the activities necessary for community reintegration were covered by the questionnaire. Each participant was expected to answer ‘yes’ or ‘no’ to each of the questions above. The percentage ‘yes’ and ‘no’ on the cognitive debriefing checklist for each question was then calculated and presented to the expert panel committee on a second meeting. Items with less than 80% positive answers were supposed to be amenable to changes. However, all the items had 100% positive answers. No modification was therefore made on the questionnaire by the panel of experts. The final Igbo version of RNLI (I- RNLI) was thus produced.

#### Phase 3 -- validation phase

The I-RNLI and the E-RNLI were either self-administered or interviewer-administered (based on participant’s preference) to 102 individuals with mobility-restricting physical disabilities (who met the inclusion criteria) from the selected centres. The essence of administering the E-RNLI was to determine the concurrent validity of the I-RNLI. A sample size of 99 had 87% power to detect a moderate change [40] at an alpha level of significance of 0.05. Sample size was calculated using G* Power 3.0.10 [41]. The order of the administration of the two questionnaires was randomized using simple randomization method. Participants who picked ‘I’ responded to the I-RNLI first while those who picked ‘E’ responded to the E-RNLI first.

### Data analysis

All analyses were conducted using the Statistical Package for Social Sciences (SPSS) version 21. The demographic and clinical variables (age, gender, level of education, marital status, occupational status, place of residence, state of origin and causes of disability) as well as the scores from the I-RNLI and the E-RNLI were summarised using frequency counts and percentages, median, mean and standard deviation. The Spearman rank order correlation and scatter plot were used to estimate the level of correlation between participants’ scores on the I-RNLI and the E-RNLI (in order to determine the concurrent validity of the I-RNLI). Bland-Altman plot was used to determine the homoscedasticity of the total scores on the I-RNLI and the E-RNLI. The Cronbach’s alpha was used to determine the internal consistency of the I-RNLI. The standard error of mean (SEM) and the minimal detectable difference (MDD) of the total, subscale and domain scores on the I-RNLI were calculated. The MDD was calculated using the following formula: MDD = 1.96 x SEM x √2 [14].

Principal component analysis (PCA) was used to determine the structural validity of the I-RNLI. The Kaiser-Meyer-Olkin (KMO) and the Barlett’s test of sphericity were used to assess the data for suitability for factorial analysis before performing the PCA. The Kaiser-Meyer-Olkin (KMO) value exceeding the recommended value of 0.6 [42] and a significant Barlett’s test of sphericity would support the factorability of the data [43]. When the coefficients of correlation of each of the items on the I-RNLI with one another all exceeded the recommended value of 0.3, it would reveal that all the items measured the same construct. During PCA, any factor with its eigenvalues exceeding 1 would be retained. The number of retainable factors could also be revealed through the scree plot by counting off the number of points before a clear point of reflection. The retained factors would then be further investigated using the Cattel’s scree plot [44]. Any component which initial eiginvalue would be lower than the random eigenvalue would be rejected. Oblimin rotation was used to further interpret the accepted components. Communality values of less than 0.3 might be indicating that the item did not fit well with the other items loading on the same component**.** Variables/items loading substantially on only one component are usually retained as part of the component. The alpha level was set at 0.05.

## Results

### Cross-cultural adaptation process of the RNLI into Igbo culture and environment

All the 11 items on the E-RNLI were retained on the I-RNLI but some modifications were made on some (three) of the items during the process of cross-cultural adaptation. Table 1 summarises the modifications. In item one, the term “living quarters”, which means ‘housing available for people to live in’ was interpreted as “compound” (comprising of “living quarters” and the immediate surroundings). In item six, there was no exact Igbo equivalent word for “recreational activities”, and this was replaced with the phrase that means “participating in activities for the sole aim of deriving joy.” This is an expression that is nearest in meaning to “recreational activities” in Igbo language. To further reduce ambiguity, the expert panel suggested that the phrase, “recreational activities” still be retained in parenthesis in the I-RNLI. “Hobby”, which was given as example of recreational activities in the E-RNLI was scrapped from the Igbo version. In item eleven, “life events” was replaced with a phrase that literally means “whatever the day brings” but contextually means “life events”. This is because the direct translation of “life events” in Igbo Language is awkward and ambiguous.

**Table 1:** Summary of words and phrases modified in adapting the RNLI to Igbo culture and environment

Item	original version	Adapted version
1	around my living quarters	Around my compound
6	participate in recreational activities (hobbies, crafts, sports, reading, television, games, computers etc.)	participate in activities for the sole aim of deriving joy (crafts, sports, reading, television, games, computers etc.)
11	deal with life events	deal with whatever the day brings

KEY:

RNLI = Reintegration to Normal Living Index.

Thirty individuals with mobility disability (18 males and 12 females) with mean age of 47.90 ± 24.38 years participated in the pretesting and cognitive debriefing interview of the present study. All the participants indicated clarity of language and ease of understanding of all the items during cognitive debriefing interview. The participants also agreed that all the activities in the I-RNLI were normally practiced in Igbo culture and therefore were relevant for community reintegration among Igbos.

### Validation of the Igbo version of the RNLI

One hundred and two individuals (61.8% males and 38.2% females) with mobility-restricting disabilities with mean age of 46.92 ± 20.91 years participated in the psychometric testing of the I-RNLI. The description of the participants is displayed on Table 2. The median disability duration among the participants was 96.00 months. The correlation between the scores obtained on the E-RNLI and the I-RNLI was used to assess the concurrent validity of the I-RNLI.

**Table 2:** Socio-demographic variables of individuals with mobility disabilities

Variables	Class	Frequency	Percentage
State of Origin	Anambra	71	69.6
	Enugu	21	20.6
	Ebonyi	5	4.9
	Imo	3	2.9
	Delta	1	1.0
	Abia	1	1.0
Residence	Anambra	79	77.5
	Enugu	23	22.5
Gender	Male	63	61.8
	Female	39	38.2
Occupation	Occupationally inactive	43	42.2
	Civil/Public/Private	7	6.9
	Students	26	25.5
	Self employed	26	25.5
Level of Education	Primary	49	48.0
	Secondary	32	31.4
	Tertiary	21	20.6
Marital Status	Married	55	53.9
	Single	40	39.2
	Divorced/separated	1	1.0
	Widowed	6	5.9
Causes of disability	Stroke	19	18.6
	Post-polio syndrome	28	27.5
	Severe osteoarthritis	13	12.7
	Leprosy	14	13.7
	Neuropathy	2	2.0
	Amputation	8	7.8
	Spinal cord injury	9	8.8
	Mal-united fracture	6	5.9
	Foot drop	2	2.0
	Osteogenesis imperfecta	1	1.0

### Concurrent validity of the Igbo version of the RNLI

**Fig. 1:**
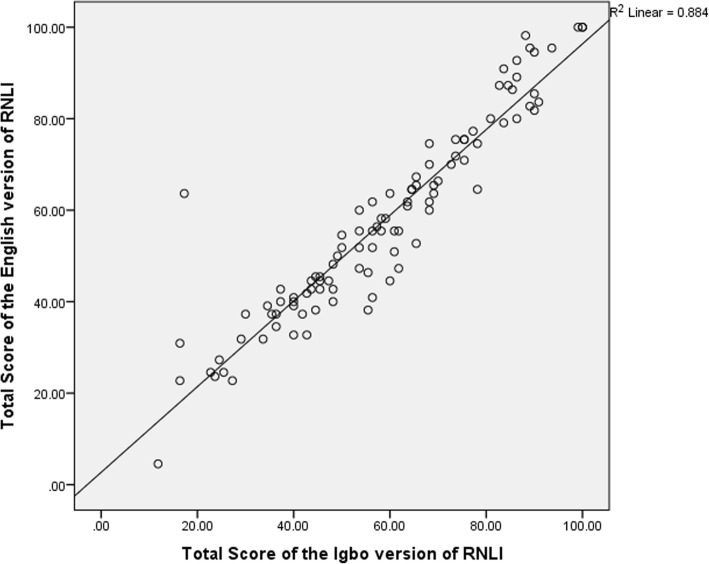
Scatter diagram for total scores on the E-RNLI and the I-RNLI. **KEY**: **I-RNLI** = Igbo version of Reintegration to Normal Living Index. **E-RNLI** = English version of Reintegration to Normal Living Index

**Fig. 2:**
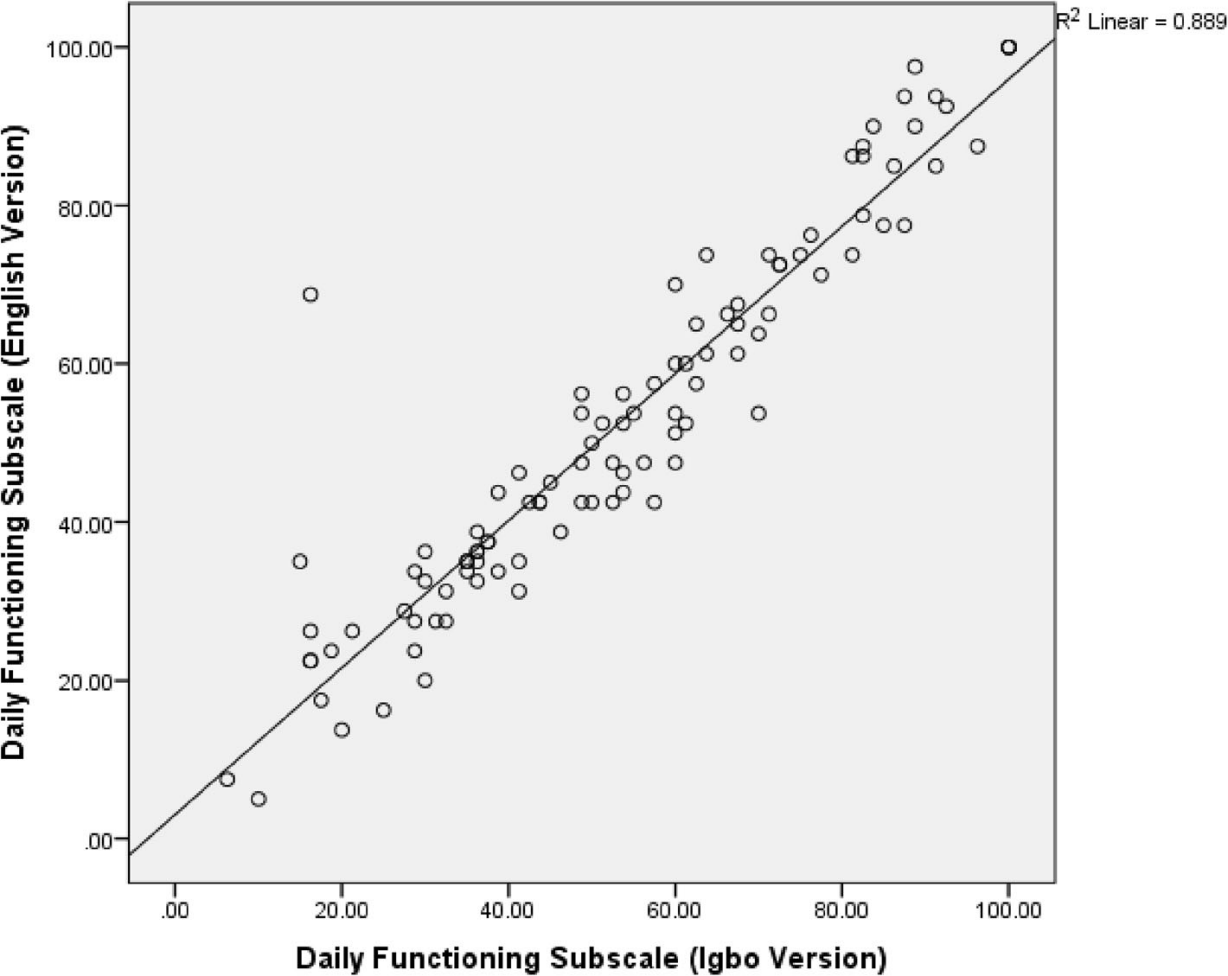
Scatter Diagram for daily functioning subscale scores on the E-RNLI and the I-RNLI. **KEY**: **I-RNLI** = Igbo version of Reintegration to Normal Living Index. **E-RNLI** = English version of Reintegration to Normal Living Index

**Fig. 3:**
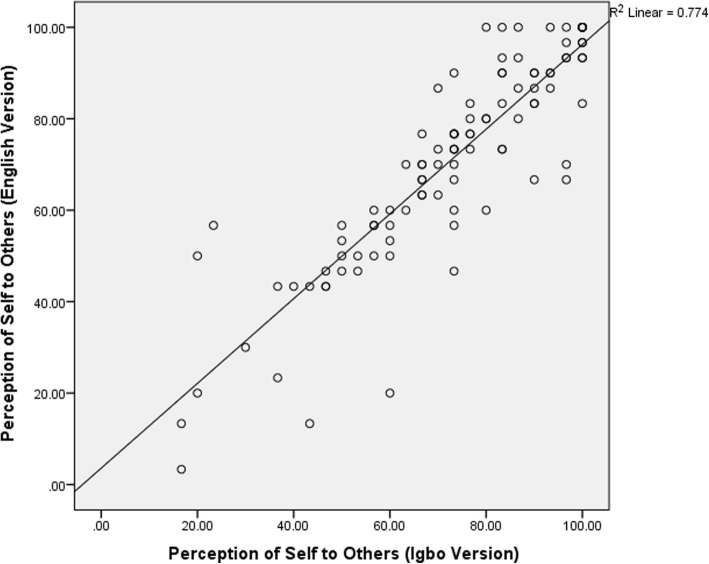
Scatter Diagram for perception of self to others subscale scores on the E-RNLI and the I-RNLI. **KEY**: **I-RNLI** = Igbo version of Reintegration to Normal Living Index. **E-RNLI** = English version of Reintegration to Normal Living Index

**Fig. 4:**
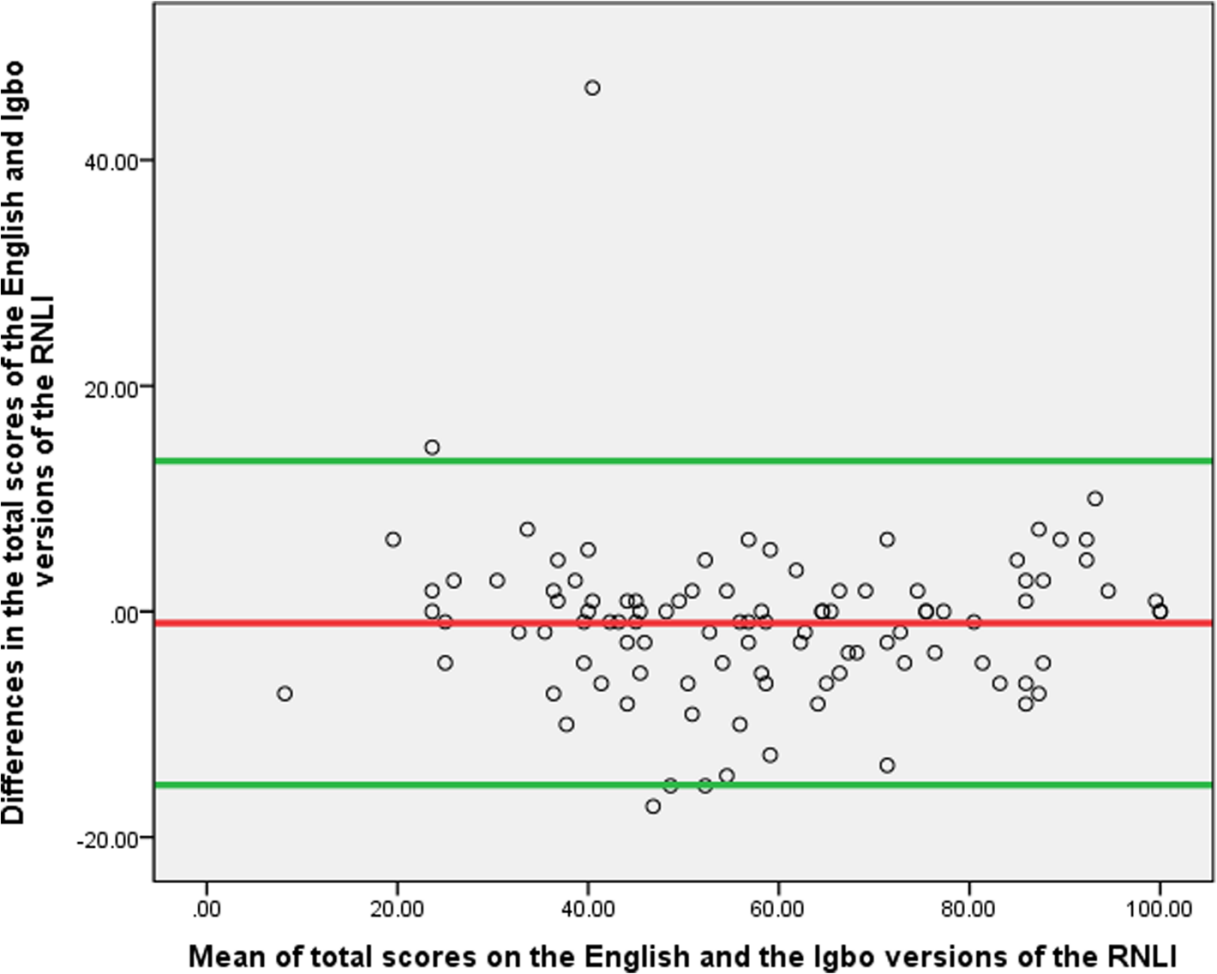
Bland-Altman plot of the total scores on the the E-RNLI and the I-RNLI. **KEY**: **I-RNLI** = Igbo version of Reintegration to Normal Living Index. **E-RNLI** = English version of Reintegration to Normal Living Index

There were significant correlations between the participants’ total, domain and subscale scores on the I-RNLI and the E-RNLI (0.81–0.95) indicating evidence of excellent concurrent validity in all the scores on the Igbo version (Table 3). The total and the “presentation of self to others” scores had the highest and the least validity coefficients respectively (Table 3). The scatter plots of the correlation between the total, ‘daily functioning’ and ‘presentation of self to others’ community reintegration scores on the two versions of the questionnaire are shown on Figs. 1, 2 and 3. Bland-Altman plot of the total scores on the I-RNLI and the E-RNLI revealed evidence of homoscedasticity of the two scores as 97% of the points fell within the 95% confidence interval (Fig. 4). The test of difference (Mann-Whitney U test) between the scores on the two versions of the questionnaire revealed no significant difference (*p* = 5.39–0.99). The mean total scores on the E-RNLI (57.60 ± 21.17) and the I-RNLI (58.62 ± 21.25) are very similar thereby supporting the linguistic and conceptual equivalence of the I-RNLI and the E-RNLI.

**Table 3:** Spearman rank correlation between I-RNLI and E-RNLI scores

RNLI Scores	R	P
Indoor mobility (item 1)	0.89	< 0.001
Community mobility (item 2)	0.85	< 0.001
Distance mobility (item 3)	0.87	< 0.001
Self care (item 4)	0.87	< 0.001
Daily activities (item 5)	0.88	< 0.001
Recreational activities (item 6)	0.82	< 0.001
Social activities (item 7)	0.84	< 0.001
Family roles (item 8)	0.87	< 0.001
Personal relationship (item 9)	0.84	< 0.001
Presentation of self to others (item 10)	0.81	< 0.001
General coping (item 11)	0.88	< 0.001
Daily functioning transformed	0.94	< 0.001
Perception of self transformed	0.88	< 0.001
Total transformed	0.95	< 0.001

KEY:

I-RNLI = Igbo version of Reintegration to Normal Living Index; E-RNLI = English version of Reintegration to Normal Living Index; RNLI = Reintegration to Normal Living Index

### Reliability analysis

The Cronbach’s alpha for the item-to-total correlation on the **I-RNLI** was 0.84, while those for split-half reliabilities for the ‘daily functioning’ and ‘perception of self to others’ subscale scores were 0.82 and 0.92 respectively. These values indicate excellent internal consistency of the items on the I-RNLI. The standard error of mean and minimum detectable difference values for the total, subscale and domain scores on the I-RNLI are displayed on Table 4. The SEM values ranged from 0.23 (out of 10) (item 9) to 2.10 (out of 100) (perception of self subscale and total scores) while the MDD values ranged from 0.63 (out of 10) (item 9) to 5.83 (out of 100) (total scores).

**Table 4:** Standard error of mean and minimum detectable difference of I-RNLI scores

RNLI Scores	SEM	MDD
Indoor mobility (item 1)	0.29	0.80
Community mobility (item 2)	0.31	0.85
Distance mobility (item 3)	0.32	0.89
Self care (item 4)	0.25	0.68
Daily activities (item 5)	0.32	0.88
Recreational activities (item 6)	0.26	0.73
Social activities (item 7)	0.26	0.72
Family roles (item 8)	0.29	0.81
Personal relationship (item 9)	0.23	0.61
Presentation of self to others (item 10)	0.24	0.67
General coping (item 11)	0.25	0.70
Daily functioning transformed	2.33	6.47
Perception of self transformed	2.10	5.83
Total transformed	2.10	5.83

KEY:

I-RNLI = Igbo version of Reintegration to Normal Living Index

### Structural validity of the Igbo version of the RNLI

The Kaiser-Meyer-Olkin (KMO) value (0.881) exceeded 0.6, which is the recommended value [Kaiser, 1970], while the Barlett’s test of sphericity was statistically significant (χ^2^ = 854.34; *p* < 0.001) thus supporting the factorability of the data. Except for the correlation coefficients between items 1 and 11 (r = 0.28; *p* = 0.003), the coefficients of correlation of each of the items on the I-RNLI with one another all exceeded the recommended value of 0.3, thus revealing that all the items measure the same construct.

#### Part 1 (PCA)

**Fig. 5:**
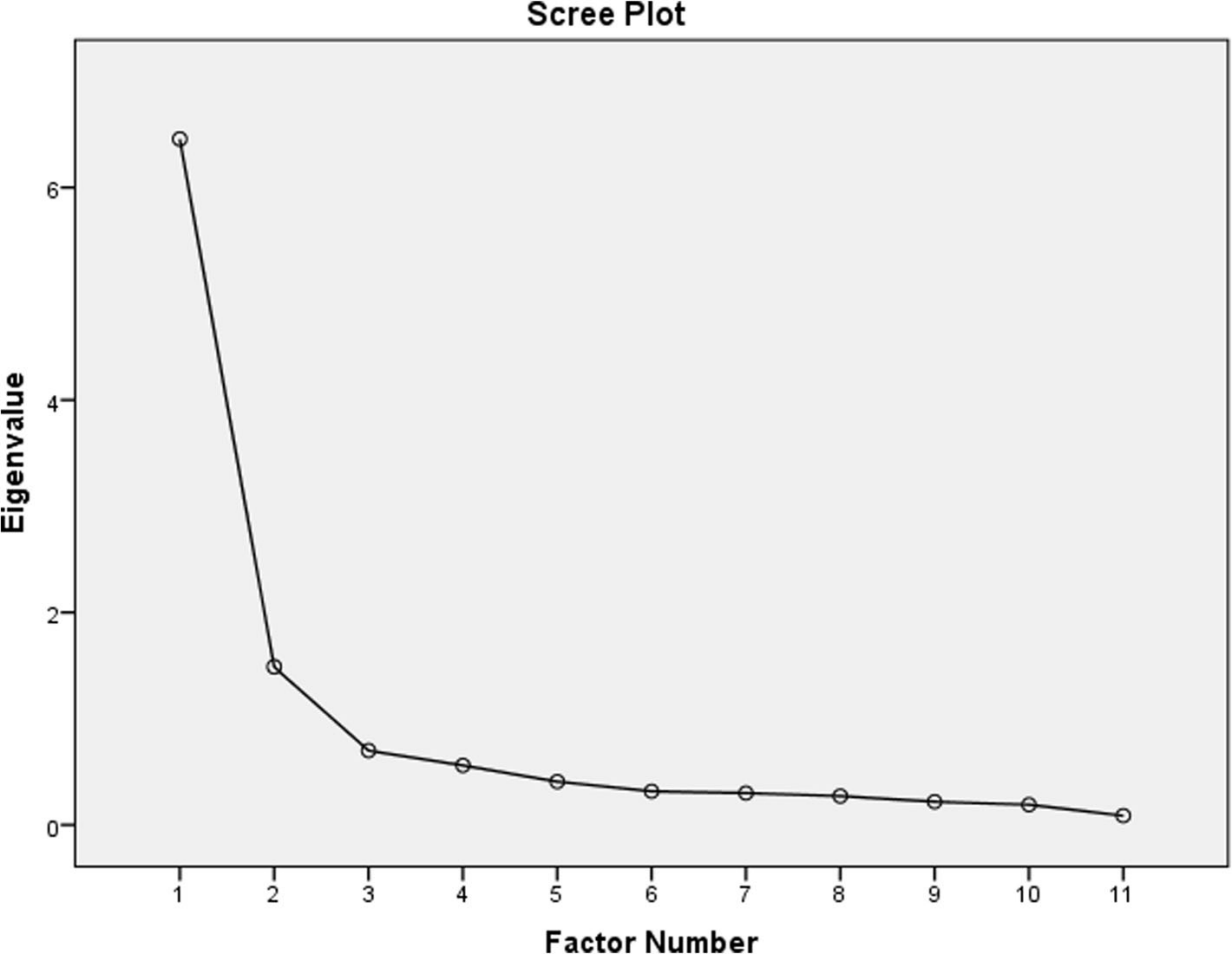
Scree plot of items on the I-RNLI. **KEY**: **I-RNLI** = Igbo version of Reintegration to Normal Living Index

The analysis revealed that there were two factors with their eigenvalues exceeding 1, explaining 58.71 and 13.53 of the variances respectively. The two components thus explained a total of 72.24% of the variances (Table 5). Scree plot also upheld the presence of these two factors by revealing a clear point of inflection after the second factor (Fig. 5). These two components were then retained for further investigation using Cattel’s scree plot. The initial eigenvalues of the two components were both higher than the random eigenvalues, and were then accepted. Oblimin rotation revealed the presence of a simple structure with both components showing a number of strong loadings and all variables loading substantially on only one component. A strong correlation existed between the two components (r = 0.55) thus supporting the use of the components as separate scales.

**Table 5:** Principal Component analysis and Monte Carlo PCA for parallel analysis of the I-RNLI

Component	Initial Eigen values	Random Eigen values	Decision	Variances%	Cumulative%
C1	6.46	5.93	Accepted	58.71	58.71
C2	1.49	1.39	Accepted	13.53	72.24
C3	0.70		Rejected	6.37	78.61
C4	0.56		Rejected	5.10	83.72
C5	0.41		Rejected	3.71	87.43
C6	0.32		Rejected	2.88	90.31
C7	0.30		Rejected	2.73	73.03
C8	0.27		Rejected	2.47	95.50
C9	0.22		Rejected	1.98	97.49
C10	0.19		Rejected	1.73	99.21
C11	0.09		Rejected	0.79	100.0

Key

RNLI = Reintegration to Normal Living Index.

#### Part 2 (Oblimin rotation of two-factor solution

A strong correlation existed between the two components (r = 0.55). The structure (correlation between variables and components) and pattern (loading of each item on the components) matrices are displayed on Table 6. The items that had the highest loading on the first component (daily functioning) were 2, 3, 1, 5, 8 and 6 in that order. The items that had the highest loading on the second component (perception of self) were 9, 10, 11, 7 and 4 in that order. Communality values of less than 0.3 might be indicating that the item does not fit well with the other items loading on the same component. However, the communality values of all the items were all above 0.3 indicating that all the items fit well.

**Table 6:** Pattern and structural matrix for PCA with Oblimin rotation of two-factor solution of the I-RNLI items

I-RNLI	Pattern coefficient		Structure coefficient		Communalities	
Items	C1	C2	C1	C2	Initial	Extraction
1	0.840		0.852	0.483	1.000	0.727
2	0.985	− 0.115	0.934	0.448	1.000	0.879
3	0.911		0.868	0.422	1.000	0.757
4	0.273	0.587	0.596	0.737	1.000	0.595
5	0.867	0.106	0.889	0.516	1.000	0.791
6	0.515	0.414	0.742	0.697	1.000	0.670
7	0.255	0.682	0.629	0.822	1.000	0.721
8	0.673	0.187	0.776	0.557	1.000	0.627
9	−0.116	0.958	0.411	0.895	1.000	0.810
10		0.882	0.435	0.855	1.000	0.733
11		0.801	0.432	0.797	1.000	0.636

Key

I-RNLI = Igbo version of the Reintegration to Normal Living Index.

## Discussion

The present study was designed to cross-culturally adapt and validate the Igbo version of the RNLI (I-RNLI) among Igbo individuals with mobility disability in Southeast Nigeria following a systematic standardized approach [26]. At the end, the I-RNLI displayed excellent concurrent validity, structural validity and internal consistency. Igbo is one of the three major native languages in Nigeria (mainly South-Eastern part) and a minor language in the Equatorial Guinea. It has about 24 million speakers with over 20 dialects. There is however a standard Igbo dialect known as Central Igbo that is well understood across all Igbo-speaking regions and by almost all Igbo-speaking individuals [35]. All the participants involved in the pretesting and cognitive debriefing interview in the present study displayed clear understanding of all the items on the I-RNLI.

A poor translation process can lead to significant differences in the original and translated versions of questionnaires thus giving erroneous comparisons of results across different translations. As a result, a widely accepted and standardized protocol [26] for translation and cross-cultural adaptation of questionnaires was adopted for use in this study. Involvement of two different translators at each of the forward and backward translations; making sure that one of the forward translators had no medical background; blinding of three of the translators about the concept of the questionnaire; and review by expert panels, were all geared towards reducing bias in the translation as much as possible. All the items on the E-RNLI were judged by the expert panel to be relevant for measuring community reintegration among Igbo individuals with mobility disabilities living in the South-eastern Nigeria. However, following the recommendations by Beaton et al. [26], some modifications were made on some of the items in order to ensure semantic, experiential and conceptual equivalence of the terms and examples in Igbo environment. In item one, the term ‘living quarters’ was replaced with ‘compound’. This in accordance with the suggestion by Beaton et al. [26] that experiential equivalence should be ensured between the original and target languages during cross-cultural adaptation. In Igbo land, greater importance may be attached to being able to move around the compound than being able to move about inside the house. In item six, ‘recreational activities’ was translated as ‘activities done for the sole aim of deriving joy’ while ‘hobby’ which was given as example of recreational activities in the E-RNLI was scrapped from the I-RNLI. The absence of exact Igbo equivalent word for ‘recreational activities’ necessitated the used of the best descriptive phrase to portray what was meant by ‘recreational activities’. ‘Hobby’ was scrapped because there was no Igbo equivalent word for it, and it was unanimously agreed by members of the expert panel that “hobby” had been fully captured in the phrase participating in ‘activities done for the sole aim of deriving joy’. In the cross-cultural adaptation guidelines provided by Beaton et al. [26], idioms, phrases or any art of speech could be employed wherever possible to relay the exact meaning of an item, construct or variable to the targeted audience. This is also why ‘life events’ in item eleven was replaced with an idiom which is literally translated as ‘whatever the day brings’. The literal meaning of ‘life events’ in Igbo Language is very awkward and ambiguous thus necessitating the use of an idiom that exactly captured its meaning in Igbo Language.

There were significant and excellent correlations between the participants’ total, domain and subscale scores on the I-RNLI and their corresponding scores on the E-RNLI, suggesting an excellent concurrent validity of the I-RNLI. This suggests that the two versions of the RNLI (the I-RNLI and the E-RNLI) are contextually equivalent and that the I-RNLI is a valid questionnaire for individuals with mobility disabilities in the Southeast (Igbo-speaking) region of Nigeria. The validity coefficients in the present study are similar to those from original and Chinese versions of the RNLI [14, 45] on validation of the RNLI. The lack of significant difference in the scores on the I-RNLI and the E-RNLI indicates that the I-RNLI was excellently translated and culturally adapted to Igbo culture and environment. All these suggest that the I-RNLI is a valid and an acceptable instrument for use in assessing the level of community reintegration among Igbo individuals with mobility disabilities in Igbo land. This result supports the alternate hypothesis that stated that the scores on the I-RNLI and those on the E-RNLI would be of significant correlation when applied to Igbo people with mobility disabilities.

The internal consistency coefficient (α = 0.84) of the I-RNLI as measured with the Cronbach’s alpha was excellent. This value is similar to those from the original and Chinese versions of the RNLI [14, 45]. This suggests that the items on the I-RNLI are homogenous and are all assessing different aspects of the same construct which is community reintegration. This supports the alternate hypothesis that the items on the I-RNLI would show significant internal consistency (homogeneity). However, it should be noted that a too-high alpha value may suggest that some items are redundant as they are testing the same question but in a different guise [46]. Streiner [47] recommended a maximum alpha value of 0.90. The SEM and MDD values of I-RNLI were also established in the present study. The MDD values reported here will be useful in the future in helping in determining whether an intervention study has induced any real change in satisfaction with community reintegration among individuals with mobility disabilities [14].

The PCA, which is intimately involved with question of validity and is usually at the heart of the measurement of psychological constructs [48], was used in determining the factor structure of the I-RNLI. PCA, as against exploratory factor analysis was chosen because the scale had already been established on an existing theory by the original authors of the scale. The revealing of two primary domains of the RNLI-I was consistent with previous studies on the RNLI [14, 45, 49]. However, the interpretation of the components varies from those of other previous studies. The two components in the present study were interpreted as “instrumental activities of daily living” (items 1–3, 5,6 and 8) and “self care, family socialisation and presentation of self to others” (items 4, 7, 9–10) contrary to the interpretations (“daily functioning” (items 1–8) and “perception of self to others” (items 9–10)) from the original and Chinese versions of the RNLI [14, 45]. Stark et al. [49] interpreted their components as “social” (items 6–11) and “physical” (items 7–11). These discrepancies in factor structure across the different studies can be adduced to some factors. The studies varied considerably in one or more of their sample characteristics, methods of questionnaire administrations, nature of the questionnaire scaling and type of statistical analysis, which have all been reported to influence results [14, 49]. In the present study, the visual-analogue-scale version of the RNLI was interviewer-administered to participants with highly varied conditions (including neurological and orthopaedics), and the validity was assessed using the PCA. In as much as Pang et al. [14] also used interviewer-administration, they recruited only stroke survivors that were made to respond to the 4-point scale version of the RNLI. They also employed the confirmatory factor analysis in determining their structural validity. Despite the fact that Stark et al. [49] also employed PCA in their analysis, they recruited only participants with neurological conditions who were made to respond only to the 10-point Likert-type scale version of the RNLI through self-administration.

Over half (61.8%) of the individuals who participated in this study were males. This is contrary to a previous report [2] that more females than males experience disability. This may not be unconnected to the fact that the present study is institution-based rather than community-based, and therefore might not have shown the true situations of things. Similar to a previous American report [45], post-polio syndrome was the highest cause of disability among the participants in the present study. This may be highlighting the disability burden of poliomyelitis. Almost half of the participants in the present study attained only primary level of education, and this is in line with a report by WHO [2] that people with disabilities usually have lower academic achievements.

### Limitations

The present study is not without some limitations. It was an institution- rather than a community-based study. Consequently, individuals not attending the sampled facilities were automatically excluded from the study. However, data was collected in at least one tertiary health institution and as many as possible rehabilitation facilities from each of the sampled States. This would have ensured some degrees of generalisability. Exclusion of individuals who could not understand English Language from the study might have introduced some degrees of bias. However, the researchers had no control over this as it was an inherent prerequisite of the adopted guideline. The fact that this study was restricted to only two out of the five States (with different dialects) that made up the South-Eastern Nigeria might have also biased the study. However, the usage of the Central Igbo Language which is understood by every Igbo-speaking individual was believed to have addressed this issue. The scope of the present work is limited as it concentrated only on the concurrent validity and internal consistency.

## Conclusion

The Igbo-culture adapted RNLI (I-RNLI) is a valid and reliable tool for assessing the level of community reintegration among mobility-restricting individuals in Igbo land, Southeast Nigeria. It is recommended that the E-RNLI be adapted to other major cultures in Nigeria (Hausa and Yoruba) and other countries, and that further studies should be done to determine the responsiveness, proxy-reliability and inter-rater reliabilities and normative data of the I-RNLI.

## Data Availability

The datasets used and/or analysed during the current study are available from the corresponding author on reasonable request.

## Abbreviations

BT1: The first back-translated version of the Reintegration to Normal Living Index
BT2: The second back-translated version of the Reintegration to Normal Living Index
E-RNLI: English version of the Reintegration to Normal Living Index
I-RNLI: Igbo version of the Reintegration to Normal Living Index
MDD: Minimal detectable difference
PCA: Principal Component Analysis
RNLI: Reintegration to Normal Living Index
SEM: Standard error of mean
T1: First Igbo translation of the Reintegration to Normal Living Index
T12: The synthesized or common Igbo translation of the Reintegration to Normal Living Index
T2: Second Igbo translation of the Reintegration to Normal Living Index
